# Investing in the future: lessons learnt from communicating the results of HSV/ HIV intervention trials in South Africa

**DOI:** 10.1186/1478-4505-9-S1-S8

**Published:** 2011-06-16

**Authors:** Sinead Delany-Moretlwe, Jonathan Stadler, Philippe Mayaud, Helen Rees

**Affiliations:** 1 Reproductive Health & HIV Research Unit, University of the Witwatersrand, South Africa; 2 Department of Infectious & Tropical Diseases, London School of Hygiene & Tropical Medicine, UK

## Abstract

**Background:**

Communicating the results of randomised controlled trials may present challenges for researchers who have to work with communities and policy-makers to anticipate positive outcomes, while being aware that results may show no effect or harm.

**Methods:**

We present a case study from the perspective of researchers in South Africa about the lessons learnt from communicating the results of four trials evaluating treatment for herpes simplex virus type 2 (HSV-2) as a new strategy for HIV prevention.

**Results:**

We show that contextual factors such as misunderstandings and mistrust played an important role in defining the communications response. Use of different approaches in combination was found to be most effective in building understanding, credibility and trust in the research process. During the communication process, researchers acted beyond their traditional role of neutral observers and became agents of social change. This change in role is in keeping with a global trend towards increased communication of research results and presents both opportunities and challenges for the conduct of future research.

**Conclusions:**

Despite disappointing trial results which showed no benefit of HSV-2 treatment for HIV prevention, important lessons were learnt about the value of the communication process in building trust between researchers, community members and policy-makers, and creating an enabling environment for future research partnerships.

## Background

Randomised controlled trials are considered the gold standard for evaluating the effectiveness of new interventions. Communicating the results of these trials may present challenges for researchers who need to work with stakeholders to prepare for potentially positive outcomes, while at the same time living with the knowledge that trials may show no effect, or even harm.

International development agencies are placing increasing emphasis on the need to communicate research results to policy-makers. In rational models of policy development, researchers conduct research to provide evidence to guide policy change. Once the research is completed, there is an expectation that policy-makers will either accept the evidence or that informed advocates will use the evidence to lobby for policy change. In this model, researchers are traditionally regarded as neutral observers. In low- and middle-income countries, there may be challenges to this model. In these settings, researchers may be influenced by the political context and be drawn into the process of advocating for policy and social change.

In this case study about communicating the results of four trials of herpes simplex virus type 2 (HSV-2) treatment for HIV prevention in South Africa, we reflect on the interactions between researchers and society, and the role of researchers as agents of social change.

## Methods

This case study was written from a researcher perspective. It represents the accumulated experience, reflections and discussions of the authors over the life of these four trials. All the authors were involved in different ways in the results communication process, and have first-hand experience of presenting these results to the different audiences outlined in the paper.

A first draft of this paper was presented at a workshop on Research-to-Policy processes in Liverpool, United Kingdom in May 2009. Earlier drafts which described the communications process were presented at investigators’ meetings for the multi-centred trials. Feedback from participants at these meetings was used in the development of this paper. In addition, the authors referred back to research dissemination plans drafted prior to trial completion, as well as reports written by staff which recorded various aspects of the results communication process, including reactions to the process by stakeholders.

### Setting and context

South Africa has one of the highest rates of new HIV infections in the world, despite over a decade of investment in primary prevention [1]. Combating HIV remains an important Millennium Development Goal yet to be achieved [2]. The high incidence of HIV, along with a relatively well-developed infrastructure, and a new commitment to human rights culture provide the ideal environment for research into new HIV prevention interventions. Despite this, there are several challenges to conducting research in this context.

The apartheid history of South Africa has left a legacy of suspicion and mistrust of research. At the centre of this suspicion is the recurring theme of conspiracy against Africans, either from the country’s white conservatives or from the pharmaceutical industry [3,4]. With respect to HIV/AIDS, this is articulated in the notion that the AIDS epidemic was conceived as a plot of the apartheid government to eradicate the black population [4,5]. While conspiracy theories may seem unfounded, they have their genesis in historical reality. It has been reported that in the final days of the apartheid regime, government laboratories were involved in attempting to spread HIV through a network of infected sex workers, amongst other things [3]. In the past decade, the debate over the origins of AIDS has highlighted the longstanding tensions between the scientific method, espoused by modern biomedical models, and indigenous knowledge systems. These tensions are best evidenced by the regular clashes between the Treatment Action Campaign and the Minister of Health, but extend throughout South African society [6].

Another legacy of apartheid is a skills shortage. This is evident in government where there is insufficient experience and skill to interpret and use research evidence to inform policy at local, provincial and national levels of government. Most communities in South Africa are relatively research-naïve. Those communities where research is conducted are often poor, have limited access to education and are experiencing the brunt of the burden of HIV. Although they may be suspicious of research, they recognise the need to respond to HIV and see research as one way to do this. However, these communities often have very little experience or insight into the research process and may have unrealistic expectations of what can be achieved. The media is frequently unable to assist or contribute constructively to the debate, and in some instances may fuel suspicion and mistrust of research [7].

In this context it is not surprising that researchers are frequently drawn into mediating these tensions between stakeholders, rather than playing the more traditional role of a “neutral observer”. In establishing and implementing HIV prevention trials in South Africa, researchers are required to build trust and address the historical stereotype of researchers as villains. They also have to manage the popular perception of biomedical certainty, i.e., that that modern medicine has all the answers. They are required to explain the scientific method and manage the expectations of sometimes desperate communities. They need to anticipate the outcomes of these trials, and support policy-makers to do the same so that research results can be rapidly translated into programmes. At the same time, they must recognise and work within the limitations of an ailing health service.

### Description of the trials

Four trials evaluating treatment for herpes simplex virus type 2 (HSV-2) were conducted in Johannesburg from 2004-2008. The trials evaluated different treatment strategies (episodic therapy or suppressive therapy using the antiherpetic drug acyclovir) and involved populations (men, women, couples) who were either HIV uninfected or HIV infected and were designed to answer a range of questions about the potential role of herpes treatment for HIV prevention (see table 1). These trials were developed following observational data that HSV-2 increases the risk for HIV acquisition [8], and that in dually infected individuals, HSV-2 infection increases HIV replication and potentially transmission [9]. However, randomised controlled trials were required to test this hypothesis using an experimental design in order to prove the causal relationship between HSV-2 infection and HIV transmission. In South Africa, all these trials were submitted through the usual regulatory processes and approved by the University of the Witwatersrand Human Research Ethics Committee and the Medicines Control Council of South Africa.

**Table 1:** Summary of design and outcomes of trials investigating the role of treatment for herpes simplex virus type 2 (HSV-2) for HIV prevention

Author	Study design & population	Intervention	Expected Outcome
**Trials of HSV-2 suppressive therapy**			

Celum et al., 2008	Randomised, double blind, placebo-controlled trial in 3172 HIV-negative, HSV-2–positive participants (1358 women, 1814 men who have sex with men)	Acyclovir, 400 mg twice daily, for 12–18 months	Prevention of HIV acquisition
Delany et al., 2009	Randomized, double-blind, placebo-controlled trial in 299 HIV-positive, HSV-2–positive women with CD4 > 250 not on HAART in South Africa	Acyclovir, 400 mg twice daily, for 3 months	Reduction of HIV infectiousness
Celum et al., 2009	Randomised, double blind, placebo-controlled trial in 3408 HIV-1 / HSV-2 dually-infected persons within HIV-1 serodiscordant Couples	Acyclovir, 400 mg twice daily, for 12-24 months	Prevention of HIV transmission and disease progression

**Trials of HSV-2 episodic therapy**			

Paz-Bailey et al., 2009	Randomised, double blind, placebo-controlled trial in 615 men with genital ulcer disease	Acyclovir 400mg three times a day, for 5 days	Reduction of HIV infectiousness Increased ulcer healing

## Results

### Context influenced the approach to communication

Our approach to communication was an organic process, but was shaped early on by several formative events. One event was the early closure of the male circumcision trial in Orange Farm due to evidence of efficacy. In this trial, male circumcision was shown to reduce the risk of HIV acquisition in men by 60% [10]. Despite the positive findings, the approach to communication of results to communities was modest. The delays in developing and adopting a national policy on male circumcision based on evidence obtained from South Africa illustrated the importance of anticipating a positive trial outcome and laying the foundation for rapid translation of research into HIV prevention policy and programmes.

Another important event was the highly publicised early closure of the cellulose sulphate microbicide trial. The trial was closed on the advice of the Data Safety and Monitoring Committee after an interim analysis which showed a higher number of HIV seroconversions in the microbicide arm compared to the placebo arm [11]. The ensuing negative media coverage with several sensationalist and inaccurate headlines [7,12], created fear and confusion in trial participants and their communities, as well as among South Africans more generally. The response of the Department of Health was to call for an investigation into the ethical conduct of these trials. While this action was welcomed by investigators, it fuelled suspicions in the public mind that participants were exploited and the trial was unethical, which was not correct. Throughout the furore, the voices of trial participants and communities were seldom heard, with journalists and government officials often speaking on their behalf. This experience emphasized the need to prepare staff, participants and communities for all possible trial outcomes, to work with communities to ensure that their voices are heard in future debates, and to work with experienced objective journalists when telling these stories.

A third event was the success of the civil society organisation, the Treatment Action Campaign in successfully challenging the Department of Health in the Constitutional Court to ensure access to nevirapine for all pregnant HIV-positive women to prevent the transmission of HIV from mother-to-child. The success of the mobilisation of communities behind evidence-based policies and programmes demonstrated the value of informed and engaged communities. Together these three experiences highlighted the importance of participants and communities as advocates of research, as well as of potentially successful HIV prevention technologies. So, when interactions with government officials became more difficult as a result of the influence of the Minister of Health and other AIDS denialists, our focus shifted away from engagement with policy-makers towards building stronger partnerships with communities.

### Building credibility through linkages

We established community advisory boards (CAB) in the trial sites. These were formed following an initial community consultation workshop to which representatives from a wide range of community-based organisations (CBO) identified during several mapping exercises as active in the trial site, were invited. Research staff provided explanations about the organisation, the research process and the importance of community participation in the research process. Information about the intention to conduct HIV prevention research was also provided. Thereafter, participants were asked to consult their organisations about future representation in a CAB. At a subsequent meeting, volunteers who represented either themselves or CBOs were included in the CAB. Terms of reference, agreed to by all members, guided the operation of the CAB, which was facilitated by research staff. CAB members were trained in research ethics and the specific details of the trial protocols. Monthly meetings were held to provide updates on the trials and to discuss progress and challenges. These meetings were also used to present on other research projects in the district. CAB members were able to interrogate protocols and ask probing questions of other investigators, for example, “Is it ethical to use a placebo?” or “What will you do about access to treatment after the trial?” These questions indicated a growing level of insight into the research process over time. Over time, we used multiple means of communication to explain the trials and the rationale for doing them including drama, music, radio and community events. These events provided opportunities for research staff to engage regularly with participants and community members about the trials and the rationale for doing them. Working with two local community radio stations, we developed a weekly one-hour phone-in radio show which dealt with sexual and reproductive health topics, including HIV. Through these shows the health information needs of communities were addressed, but more importantly a dialogue between the researchers and the community was established.

As the trials progressed, it became important to consider what the implications of a positive outcome would mean for future programmes. The experiences of participants in the four trials allowed us to respond to challenges from critics that daily acyclovir would not be feasible as an HIV prevention intervention. We conducted additional research which informed questions around access to drug, cost and cost-effectiveness, testing strategies and whom to target for intervention, as well as how best to ensure optimal treatment adherence. Investigators worked with international agencies such as the World Health Organisation (WHO) to inform the development of several guidelines for the use of herpes therapy. As the South African National AIDS Council (SANAC), which encompasses representatives from government and 17 sectors of civil society, was revived, investigators were also able to make presentations to this body and its committees about the trials and the need to consider the implications of a positive outcome for the National Strategic Plan and HIV prevention programmes.

As the results from other trials in Africa became available [13,14], discussions with participants and communities focussed on planning for access to acyclovir for former trial participants and ensuring access to care for those who were HIV positive. The importance of completing all the trials in order to answer all the research questions was also emphasised. For many community members, the message that these were *trials* and not health programmes only began to set in as the first of the trials closed. Seeing the experiences of others helped to focus staff and community members on planning for all possible trial outcomes. At this stage, media training for staff and CAB members by media experts helped to consolidate key messages about the trials for all possible outcome scenarios. For both staff and community members the idea that the trials might not show an effect was still a difficult one, despite the training that they had received about the nature of randomised controlled trials. This observation that staff, participants and community members were heavily invested in a positive outcome showed how important these trials were at a personal level in terms of the hope they represented to people in the fight against HIV.

### Communicating the evidence

The first of the four trials’ results from Johannesburg were available in early 2007, and the results from the final trial were released in May 2009. Over the two-year period, we developed an approach to communicating research results which included a set of activities common to all trials. However, in communicating the results of four trials this process became iterative and allowed us to modify and refine our approach based on previous experiences. The fact that the trials were examining different aspects of the interactions between HSV-2 and HIV allowed us to build a narrative that assisted with communicating these relatively complex results to stakeholders over time.

Central to the communication process was the development of a communication plan. This plan mapped out the target audiences, as well as the timing and means of communication. The plan became more sophisticated with each successive trial that was communicated. In terms of audience, three main groups with slightly different communication needs were identified, namely trial participants, the CAB and community members, and then other stakeholders who included the Department of Health, regulatory bodies such as the Research Ethics Committee (REC) and the Medicines Control Council (MCC), other interested parties including other researchers and organisations dealing with HIV, and selected health and science journalists. Timing of communication was also a critical consideration. There was an attempt to communicate the results of the trial to participants, CAB and key stakeholders in the Department of Health, the REC and the MCC at least 24 hours before the public announcement of trial results either by press release or at a conference. The purpose of this exercise was to forewarn all three groups of the results so that they were not caught unawares. In the case of the CAB and key stakeholders, this gave them time to prepare responses to the results if needed. The narrow time-frame and the need to protect the confidentiality of results during this period also determined the means of communication. For participants, communication of approved messages via short message service on cellular phones proved successful. In some cases, these messages were also delivered to CAB members, although it was easier to have face-to-face meetings with CAB members. For pre-release contacts with the Department of Health, REC and MCC, the actual results also influenced the means of communication. Generally, these groups were not available for face-to-face meetings unless there were important policy changes arising from the results. With these trials, communication was generally by telephone communication but in some cases email communication was sufficient.

After their initial public release results continued to be communicated primarily through a series of face-to-face events. Workshops were held for participants and community members where results were presented in local languages and using drawings or images to illustrate important concepts. Questions and responses were documented so that messages for subsequent workshops could be refined. Central to all the communication activities was a combined workshop, which brought together the researchers, participants and community members, the Department of Health and other researchers or organisations dealing with HIV, to discuss the trial results and the implications for policy and programmes from all perspectives. At one such event a participant spontaneously disclosed her role as a trial participant, her positive experience of the trial and the benefits of herpes treatment on her life. She was able to ask the Department of Health official what the implications of the trial would mean for access to acyclovir at primary health care clinics. For the researchers, this example best illustrated the value of engaging with participants and community members and helping them to develop the knowledge and skills to advocate for new health interventions. Another important face-to-face activity was the introduction of ‘unblinding visits’. At these visits, trial staff counselled participants on the results of the trial and their treatment allocation arm, using an ‘unblinding’ script approved by the REC. Participant responses to their ‘unblinding’ were documented in patient files. Responses were monitored and reported in internal project reports. Despite all the communication activities, the majority of participants said at the unblinding interview that they had not heard the trial results. Generally, participants accepted the information about their trial. Some participants however reported feeling disappointed or in some cases cheated. Participants reported “forgetting” that there was a placebo arm. During subsequent discussion, participants acknowledged that they recalled the informed consent discussion and did understand that there was a placebo arm, but could not believe that staff would give them the placebo. Some participants said that they expected that the placebo would look different. Some participants did not believe that they were on the placebo arm because they had experienced improvements in symptoms. Others did not believe that they were on placebo because their study drug bottle was labelled acyclovir (the label actually said “acyclovir/placebo 400 mg”). These interviews revealed the difference between participants intellectual versus their experiential knowledge of the trial [15] and illustrated the importance of personalising the trials results for the individuals, as well as reiterating concepts specific to trials like blinding, randomisation and placebo which were originally discussed during the informed consent process.

Not surprisingly, in the build up to communicating the trial results there were anxieties about dealing with the media and the media responses. In order to manage this relationship sensitivity, several actions were taken. Journalists with special interests in health or science reporting were alerted when the results were about to be released and invited to communication events. In addition, the local community radio shows that ran during the trials were used to communicate the findings over a series of shows. Training of staff and, where possible, community representatives, in handling and responding to media queries proved the most invaluable. This was best demonstrated in the most recent trial, during a pre-release meeting between investigators and CAB members. At this meeting, all questions were responded to and documented. CAB members were then asked to think of the most difficult questions they might be asked by community members or local journalists, and these were added to a list. CAB members were then paired and asked to work through the questions and their responses, using an interview format. This activity was repeated until CAB members felt comfortable with the questions and their responses. In the subsequent discussion, CAB members articulated how useful the exercise was for internalising and rehearsing the trial messages. They also articulated how activities such as these demonstrated the trust between researchers and the community.

### Impact of evidence on policy

Given all this investment in communication, the results of the trials themselves were disappointing. Overall, the trials showed that despite the observational evidence, daily acyclovir was not sufficient to prevent HIV acquisition [16]. Moreover, although two trials showed that acyclovir was able to reduce infectiousness (through reductions in plasma and genital HIV viral loads) [13,17], this was not sufficient to prevent HIV transmission [18]. An important finding from these two trials was the observation that daily acyclovir reduces plasma HIV viral load which was ultimately shown to result in modest but significant delays in HIV disease progression and death. Of value, although expected, was the finding that the trials showed an impact of daily acyclovir on the frequency of genital ulcer episodes, and in the episodic therapy trial, acyclovir improved ulcer healing by an average of two to three days [19].

While the South African trials were essential for answering important research questions, the results meant that no change in HIV prevention policy was required. Although not the primary focus in all cases, these results did influence guidelines around case management of genital ulcer disease (GUD), and acyclovir is now recommended for the first-line management of GUD at the primary health care level. New data regarding the potential use of acyclovir as an intervention to delay disease progression in HIV-positive people not yet eligible for anti-retroviral therapy did emerge and has important policy implications for HIV management. However, more data is needed to understand the financial implications and potential risks for the emergence of HSV or HIV resistance before policy is likely to be changed.

## Discussion

Despite the disappointing results, these trials were important because they demonstrated the need for using the best research designs to answer important scientific questions without ambiguity. Several lessons were learnt during the results communication process which may be of relevance for the communication of research results more generally.

The first finding is that contextual factors strongly influenced the approach to communication. In response to the mistrust and lack of understanding about research at the time, researchers engaged in an intensive approach to communication. This approach was aimed primarily at addressing what was perceived as a lack of knowledge and skills to understand and interpret the results of randomised controlled trials. Regular interactions from the start of the trials, using multiple approaches in combination, appear to have been the key to success. Using a range of communication methods ensured that the broadest possible audience was reached. Using opportunities to repeat the message through different formats helped to correct misunderstandings and reinforce the trial results. Strategies which involved personal contact like the CAB meetings, stakeholder events, or the unblinding visits were very important in the success of communication. The importance of personal contact and opportunities for two-way communication have been highlighted by other authors [20,21].

Focussing on communications from the start of the trials gave time to build understanding, as well as trust. Through text messaging, home visits, community events and the radio shows, the trials were made more visible and familiar to ordinary citizens. Through the CABs, the trials were opened up to scrutiny by community members. These strategies all made the research more accessible, built a greater understanding of the research process, and facilitated the development of more personal relationships between the researchers and the study communities. During the life of the trials, participants and community members developed a sense of ownership of the research and became invested in its successful completion, rather than in just its success. In this context, researchers could then talk about the uncertainty of trial outcomes and prepare participants and communities for these different outcomes. This preparation was critical in the subsequent acceptance by participants and communities of the actual trial results because the range of trial outcomes had been discussed, debated and understood.

The second finding is that researchers actively managed the communications process. In doing this, they became agents of social change. For the authors, while the idea of active engagement was intentional, the concept of social change was not conscious at the time. What the case study shows is that the researchers recognised that the government of the day might not act on the evidence generated from the trials. It shows that they understood that civil society responses to an intractable government were part of the South African experience. Researchers recognised that the evidence from the trials alone might not be sufficient to change policy, and community mobilisation and an informed media might be needed to ensure the translation of evidence to policy. In response, researchers stepped outside of their traditional role and actively engaged in the communication process. In so doing, they were required to respond to the legacies of apartheid, that is the lack of knowledge, the lack of skill and the lack of trust in science. In this context, knowledge is power. While the communication activities were ostensibly aimed at increasing knowledge about research, they indirectly shifted the balance of power in favour of participants and communities.

The case study demonstrates several examples where activities aimed at empowering stakeholders were attempted. Participants and communities were provided with information about the trials, including the rationale, the design, and an explanation of the key concepts of the trials as well as the results. Skills building workshops around communicating key messages from the trial gave participants and community members confidence to speak about the trial results and experience from their perspective. Through the stakeholder events as well as the radio shows, participants and community members were given access to a range of platforms that they might not otherwise have had access to, to tell their stories. Through the results communication process, groupings who might not ordinarily meet like government officials, community members and researchers were brought together to discuss and consider the trial results together. In these situations, participants and community members were given opportunities to be heard directly by policy-makers and service providers.

Government institutions and the media were also supported through these processes. SANAC, which had been weakened by the debates around the origins of AIDS, was now supported. By recognising the leadership of key government institutions and actively engaging with them, by sharing information and knowledge with politicians, government officials and regulatory authorities about the trials, researchers were able to build credibility and trust in the research results when they became public. In engaging with the media, there was recognition of the need to build a partnership with trusted journalists who could be relied upon to present clear messages about the trials and their results. By supporting a community radio station throughout the trial, community journalists were empowered to broadcast clear messages about research with local relevance.

The process of empowerment was not without challenges. The communications approach was aimed at strengthening the trial and not an end in itself. The resources available for these activities came from the trial. As such, the researchers were not in the best position to fully address the social and economic inequalities observed in the study communities. During the trials, there were times when these inequalities became a source of conflict. For example, there were situations where individual CAB members tried to hijack the trial activities for their own gain, e.g. insisting on catering contracts during participant events. Some CAB members became so invested in the trial activities that they felt they should be paid as research staff. To community members and participants, the researchers appeared to have access to money and other resources. It was almost expected that some individuals would try to take advantage of that situation. For research staff, particularly field workers who often lived in these communities, these were uncomfortable situations. In all cases, these conflicts were resolved through dialogue. Discussions focussed on the scope and limitations of the research organisation in terms of resources. Where real needs were identified, researchers used their position and networks to identify partner organisations working on poverty and development issues to assist with these needs. Including local government, religious groups and other community organisations in the CAB was helpful in this regard. It was also was interesting to observe that in situations where individuals were perceived to be acting in their own interests, good sense prevailed and other CAB members were instrumental in resolving these conflicts and re-focussing attention on the trial and the greater goal of HIV prevention, with very little intervention by the researchers. Even with this intensive approach to communications, the research teams were not able to reach all participants to communicate the research results. Although policies around the treatment of GUD changed, we were not able to influence as easily the way in which programmes were delivered. While arrangements were made for participants to have access to treatment and care after the trial, participants complained about the quality of service provided in public sector clinics.

Finally, this case study provides an opportunity to reflect more generally on approaches taken to communicating the results of clinical research. This is an emerging field, and there is very little published on this topic in relation to countries outside North America and Europe. Some authors suggest that more evidence is needed about appropriate methods for disseminating trial results to participants and the impact of these, arguing that providing results of trials to trial participants is not straightforward and constitutes an intervention in its own right [22-24]. In a recent review which summarised the effects of communicating research results on participants in 28 studies, investigators and the research enterprise, the authors noted that despite the growing number of national and international policies and guidelines concerning the duty to return research results, debate still continued over the scope and limits of investigator’s responsibilities in this regard. Communicating results was found to be highly desirable to participants and to a lesser extent investigators, but the mechanisms for doing this were poorly developed [25]. All of the studies reviewed were conducted in North America or Europe, and the majority involved cancer or genetic studies.

The HIV prevention field has been concerned with how best to protect the rights of trial participants and communities when inequalities in power, wealth, education and literacy exist between the individuals proposing to conduct research and those who are hardest hit by the HIV epidemic. For the trials reported in this case study, very little explicit official guidance was given on the communication of research results at the onset of the trials. In an updated edition of the South African Good Clinical Practice Guidelines which became available shortly before the results of the first trial in the case study were reported, investigators are regarded as having “an ethical obligation to disseminate research results, whether positive or negative, in a timely manner” [26]. Further advice suggests that results be communicated to participants and interested community members using appropriate formats but beyond that there is little guidance for investigators. The South African investigators of the cellulose sulphate microbicide trial published their experience of communicating the early closure of a trial. This provided practical insights particularly around dealing with the media [11]. In November 2007, UNAIDS published guidelines on Good Participatory Practice for Biomedical HIV prevention trials [27]. These guidelines aimed to provide systematic guidance on the roles and responsibilities of entities funding and conducting HIV prevention trials towards participants and communities. An earlier version of this document released in 2000 led to many HIV prevention trials adopting a community engagement approach. The 2007 document consolidated the experiences of many of these trials conducted in a wide range of contexts and attempts to give more specific guidance for sponsors and investigators on how to engage with communities. While this document is much more practical in its advice, concerns have been raised by investigators in low- and middle-income countries that these guidelines are aspirational and will remain so as long as resources are not provided to support community engagement in trials [28].

The resources required for the communication of research results remain a concern for investigators generally. There is little published data on the costs of communicating research results to participants. In this case study, all communication costs were covered by trial funds, in keeping with UNAIDS recommendations [27]. The largest cost item apart from staff costs was the radio show which was shared by several trials. All communication activities were conducted by trial staff as part of their trial responsibilities. We invested resources in training trial staff in communications and media handling rather than employing individuals with specialist media and communications skills. This approach made it possible for trial staff to communicate the research results in the local languages at the site, allowed for the experience of research results communication to be transferred from one project to the next and expanded the pool of individuals available to talk about the research results in a variety of settings. Even in the context of limited funds, there is a lot that can be achieved in the way of communications with a team of experienced staff. There are legitimate concerns that these activities may distract researchers from the academic expectations that they publish. While this view may be true in the short-term, the initial investments in time and energy lay the foundation for lasting productive research partnerships.

## Conclusions

Despite disappointing trial results for the field of HIV prevention, the investments in communicating research results and building partnerships were essential for building trust between researchers, communities and policy-makers, creating a more enabling environment for future research to take place.

## Competing interests

The authors declare that they have no competing interests.

## Author contributions

SDM conceived and drafted the first version of the manuscript. JS, PM, and HR provided data and/or useful insights and critically reviewed the manuscript.
